# Genome-Wide Identification of *Aquaporin* Gene Family in Pitaya Reveals an *HuNIP6;1* Involved in Flowering Process

**DOI:** 10.3390/ijms22147689

**Published:** 2021-07-19

**Authors:** Xiaoying Ye, Yongshun Gao, Canbin Chen, Fangfang Xie, Qingzhu Hua, Zhike Zhang, Rong Zhang, Jietang Zhao, Guibing Hu, Yonghua Qin

**Affiliations:** 1 Guangdong Provincial Key Laboratory of Postharvest Science of Fruits and Vegetables/Key Laboratory of Biology and Genetic Improvement of Horticultural Crops (South China), Ministry of Agriculture and Rural Affairs, College of Horticulture, South China Agricultural University, Guangzhou 510642, China; yexiaoying@stu.scau.edu.cn (X.Y.); chenjiayi98@stu.scau.edu.cn (C.C.); xie@stu.scan.edu.cn (F.X.); huaqingzhu@stu.scau.edu.cn (Q.H.); poloky@scau.edu.cn (Z.Z.); r-zhang@scau.edu.cn (R.Z.); jtzhao@scau.edu.cn (J.Z.); guibing@scau.edu.cn (G.H.); 2 Guangxi Academy of Agricultural Sciences, Nanning 530007, China; 3 Institute of Forestry and Pomology, Beijing Academy of Agriculture and Forestry Sciences, Beijing 100097, China; ysgao@scau.edu.cn

**Keywords:** pitaya, aquaporin, genome-wide identification, *HuNIP6;1*, early flowering

## Abstract

Aquaporins (AQPs) are essential membrane proteins involved in seed maturation and germination, stomata movement, photosynthesis, and regulation of plant flowering processes. Pitaya flowers are open at night and wither at daybreak, which shows an obvious circadian rhythm. In this study, a comprehensive genome-wide analysis of *AQPs* in *Hylocereus undantus* was conducted to screen key genes associated with flowering processes. A total of 33 *HuAQP* genes were identified from the *H. undantus* genome. The 33 *HuAQPs* were grouped into four subfamilies: 10 PIPs, 13 TIPs, 8 NIPs, and 2 SIPs, which were distributed on 9 out of 11 pitaya chromosomes (Chr) (except for Chr7 and Chr10). Results from expression profiles showed that *HuNIP6;1* may be involved in pitaya’s floral opening. *HuNIP6;1* was localized exclusively in the cell membrane. Overexpression of *HuNIP6;1* in *Arabidopsis thaliana* significantly promoted early flowering through regulating negative flowering regulators of *MJM30*, *COL9*, and *PRR5*, suggesting that *HuNIP6;1* plays key roles in regulating flowering time. The present study provides the first genome-wide analysis of the *AQP* gene family in pitaya and valuable information for utilization of *HuAQPs*.

## 1. Introduction

Aquaporins (AQPs) belonging to the major intrinsic protein (MIP) superfamily have been found in all types of organisms, including bacteria, fungi, animals, and plants. In 1988, a new intact membrane protein named CHIP28 was purified from erythrocyte and renal cell membrane [1]. Then, *CHIP28* was termed as *Aquaporin-1* (*AQP1*), since it showed the function of water transportation [2]. With the rapid increase in available whole genomic sequences in recent years, AQPs have been identified in various plant species, including 35 from *Arabidopsis thaliana* [3], 35 from *Zea mays* [4], 28 from *Beta vulgaris* [5], and 35 from *Citrullus lanatus* [6]. According to the homology of amino acids and subcellular location, plant AQPs are classified into five subfamilies: plasma membrane intrinsic proteins (PIPs), tonoplast intrinsic proteins (TIPs), nodulin 26-like intrinsic proteins (NIPs), uncategorized (X) intrinsic proteins (XIPs), and small basic intrinsic proteins (SIPs) [7].

Each AQP contains six transmembrane (TM) domains (TM1–TM6) connected by five loops (Loops A–E), as well as the two signature asparagine–proline–alanine (NPA) motifs located at the N-terminus of the two half-helices (HB and HE) in LB and LE [8]. The NPA motifs (signature motifs) are located inside the pore responsible for electrostatic repulsion and charge selectivity [9]. The NPA motifs make the size and hydrophobicity of the pore contraction site different, which determines the specificity of the substrate. The structural characteristics of AQPs are particularly essential to the accuracy of their functions [10].

AQPs are a kind of multifunctional membrane protein that plays an indispensable role in plant development [11]. In addition to water transport, AQPs can also transport some small solutes, gases, and metal ions across the membrane [12,13,14,15]. AQPs are involved in leaf stomatal movement [16,17], photosynthesis [18], stress-resistance effectiveness [19,20,21,22,23], and floral opening [24,25,26,27,28,29].

Pitaya, also known as pitahaya or dragon fruit, is a perennial climbing fruit crop belonging to *Hylocereus* or *Selenicereus* in the Cactaceae family. There are mainly two species of dragon fruits; i.e., white-flesh dragon fruit (*H. undatus*) and red-flesh dragon fruit (*H. monacanthus*) that have been commercially produced at a large scale as a new types of fruit crops in many countries. Their differences mainly lie in flesh color and self-pollination ability. Many species of *H.* *monacanthus* are self-incompatible [30]. Thus, artificial cross-pollination is essential to increase fruit setting and fruit weight [31]. Pitaya flowers, also called Moonflower, the Queen of the Night, and the Lady of the Night, only open at night and close at daybreak, with each flower lasting only one night [32,33]. At night, it is very inefficient, labor-intensive, and time-consuming for pitaya hand-pollination. Therefore, identification and characterization of key genes related to control of the process of floral opening in pitaya is of great significant to the pitaya industry. Although the *AQP* genes play multiple roles in various plants, the function of the *AQP* family in pitaya remains unknown. In this study, the genome-wide identification and characterization of pitaya *AQP* genes (*HuAPQs*) related to the floral opening process was performed using available pitaya genomic information [34]. The aim of the present study was to identify and validate the candidate *AQPs* genes involved in pitaya flower opening that can be used for genetic improvement of cultivated pitaya.

## 2. Results

### 2.1. Identification, Classification, Nomenclature, and Properties of HuAQPs

**Figure 1:**
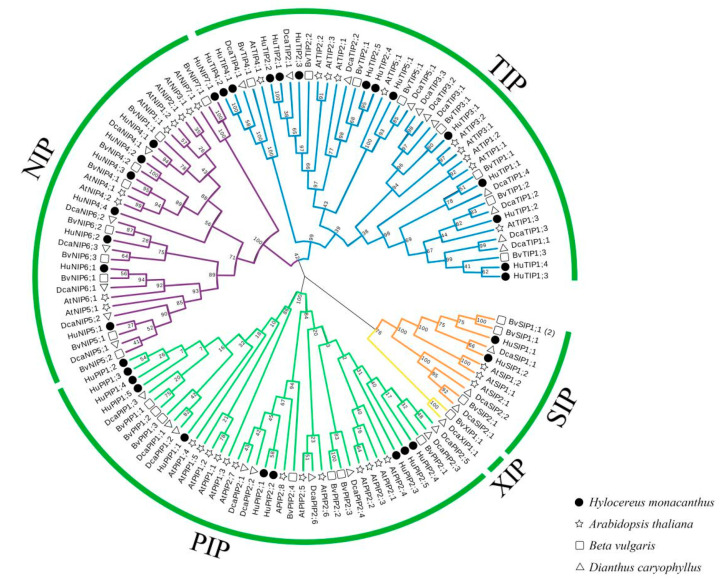
Neighbor-joining phylogenetic tree of aquaporin proteins from *Hylocereus undantus*, *Arabidopsis thaliana*, *Dianthus caryophyllus*, and *Beta vulgaris*. The phylogenetic tree was constructed using MEGAX software with a bootstrap test conducted 1000 times. AQPs were divided into five subfamilies labeled with various colors to indicate different subfamilies.

A total of 43 gene sequences were obtained in the pitaya genome database (http://www.pitayagenomic.com/) using the keyword ‘aquaporin’, and 33 *AQP* genes (*HuAQPs*) had one complete MIP domain and two NPA motifs (Table 1). Based on sequence cluster analyses of *H. undantus*, *A. thaliana*, *D. caryophyllus*, and *B. vulgaris*, those *HuAQPs* were classified into four subfamilies: 10 PIPs, 13 TIPs, 8 NIPs, and 2 SIPs (Figure 1). The PIPs subfamily included PIP1s and PIP2s subgroups, with five PIP1s and five PIP2s. The TIPs subfamily was divided into five subgroups: four TIP1s, five TIP2s, one TIP3s, two TIP4s and one TIP5. The NIPs subfamily contained four subgroups; i.e., four NIP4s, one NIP5, two NIP6, and one NIP7. HuAQPs were named according to their percentage of identity to the 35 AQP proteins from *A. thaliana* [3]. Among the 33 predicted HuAQP proteins, the largest one was *HuNIP5;1*, with 310 amino acids (AAs), while the smallest protein was *HuNIP6;2* with 217 AAs (Table 1).

### 2.2. Gene Structure and Conserved Motif Analyses of HuAQPs

**Figure 2:**
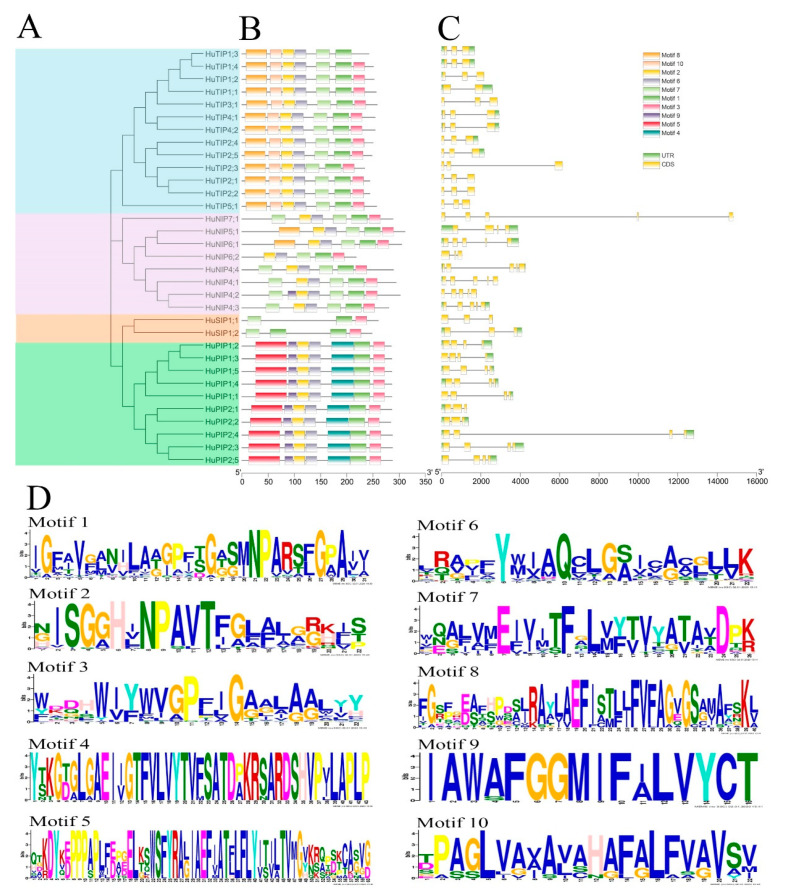
Phylogenetic relationship, gene structure, and conserved motif analyses of *HuAQP* genes. (**A**) A neighbor-joining phylogenetic tree constructed by MEGAX with 1000 bootstrap replicates using amino acid sequences of *HuAQPs*. (**B**) The motif compositions of HuAQP proteins. Ten motifs are displayed in different-colored rectangles. (**C**) Exon–intron structure of 33 *HuAQP* genes. Green rectangles represent exons, and black lines with the same length represent introns. Yellow rectangles indicate the UTR region. (**D**) The amino acid sequences of 10 motifs of HuAQP proteins.

The exon–intron structures play vital roles in plant evolution. The exon–intron structures and conserved motifs of *HuAQPs* were analyzed. Most PIP subfamilies had four exons, except for *HuPIP2;1* and *HuPIP2;2* containing three exons. TIP subfamilies had three exons, except for *HuTIP1;1* with two exons. NIP subfamilies contained five exons, while *HuNIP5;1* and *HuNIP6;2* had four exons and three exons, respectively. All SIP subfamilies contained three exons (Figure 2B). As shown in Figure 2C, Motif 1 and Motif 3 were found in all *HuAQPs*, except *HuTIP1;3* without Motif 3. Both PIP and TIP subfamilies contained seven motifs, suggesting that they were highly conserved in pitaya. Motif 5 and Motif 9 were unique to the PIP subfamilies, while Motif 10 specifically existed in the TIP subfamilies. NIP subfamilies had Motif 1, Motif 2, Motif 5, Motif 6, and Motif 7, except for *NIP6;2*.

### 2.3. Gene Mapping on the Pitaya Chromosomes

**Figure 3:**
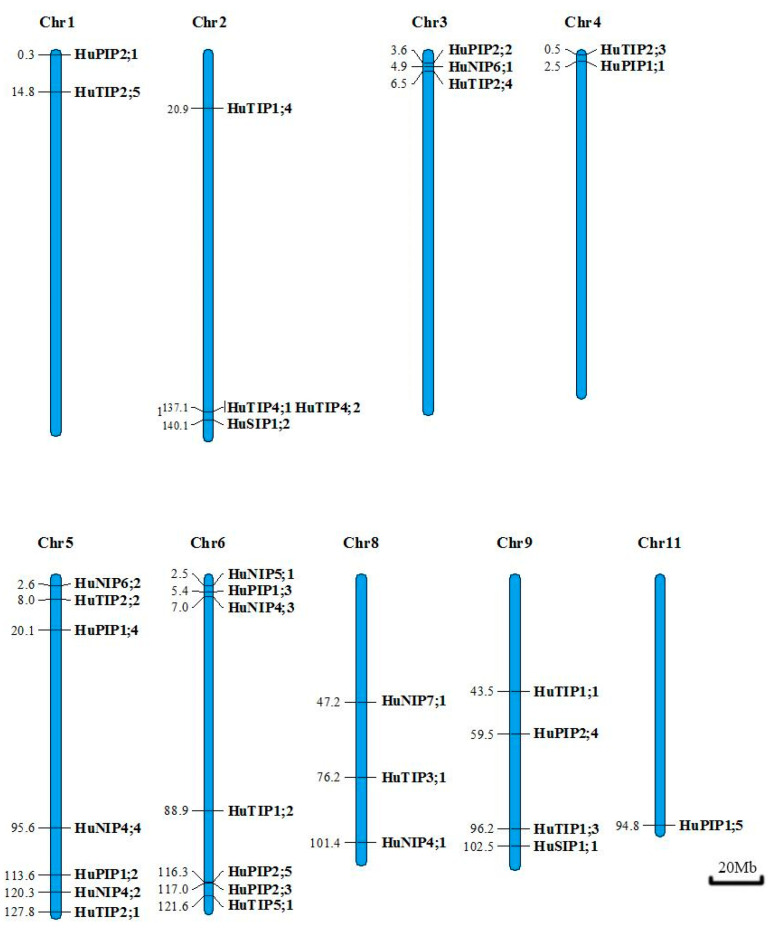
Schematic representations of the chromosomal locations of the *HuAQP* genes. The chromosome number is indicated on the top of each chromosome. Bars = 20 Mb.

According to the gene loci information, the 33 *HuAQP* genes were unevenly distributed in nine chromosomes. *HuAQPs* were mainly distributed at both ends of chromosomes, and density of *HuAQPs* varied on individual chromosomes. Most of the *HuAQPs* were located on Chr 5 (seven genes; 21.2%) and Chr 6 (seven genes; 21.2%). However, there was only one *HuAQP* on Chr 11 (Figure 3).

### 2.4. Expression Patterns of HuAQPs during the Flower-Opening Process in Pitaya

**Figure 4:**
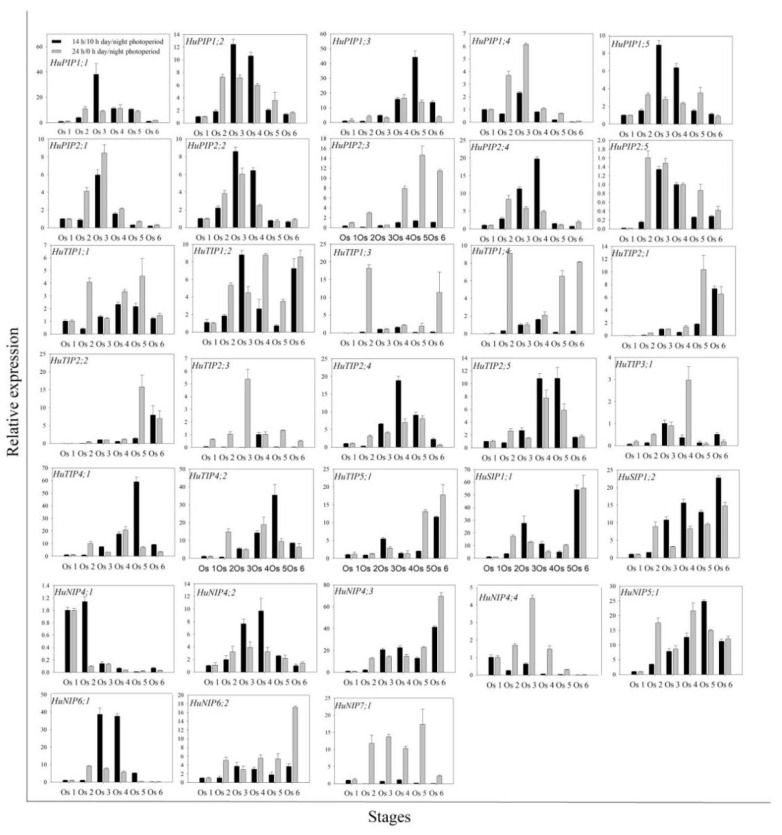
RT-qPCR analyses of *HuAQP* genes in petals during flower opening under 14 h/10 h day/night and 24 h/0 h day/night at 25 °C. The flower-opening process was separated into 6 stages, including opening stage 1 (Os 1), 12 h after culture (periods before flowering); Os 2, 24 h after culture (periods before flowering); Os 3, 28 h after culture (periods during flowering); Os 4, 32 h after culture (periods during flowering); Os 5, 36 h after culture (periods after flowering); Os 6, 40 h after culture (periods after flowering). Data represent average values from three biological replicates (±S.D.).

The expression differences of *HuAQP* genes were analyzed in petals at six flower-opening stages under 14 h/10 h day/night and 24 h/0 h day/night (Figure 4). Expression levels of *HuPIP1;1*, *HuPIP1;2*, *HuPIP1;4*, *HuPIP1;5*, *HuPIP2;1*, *HuPIP2;2*, and *HuPIP2;5* showed increasing patterns from Os 1 to Os 3, and then decreased thereafter under 14 h/10 h day/night. Similar expression patterns were also observed in *HuPIP1;4*, *HuPIP2;1*, *HuPIP2;2*, *HuTIP2;3*, *HuNIP4;2*, and *HuNIP4;4* under 24 h/0 h day/night. The highest expression levels of *HuPIP2;4*, *HuTIP1;1*, *HuTIP1;4*, *HuTIP2;3*, *HuTIP2;4*, *HuTIP2;5*, and *HuNIP4;2* were detected at Os 4 under 14 h/10 h day/night, compared to the highest expression levels of *HuPIP1;3* and *HuTIP3;1* at Os 4 under 24 h/0 h day/night. The expression of *HuTIP4;1*, *HuTIP4;2*, and *HuNIP5;1* reached their maximums at Os 5 under 14 h/10 h day/night, while the expression of *HuTIP2;1*, *HuTIP2;2*, and *HuTIP2;4* peaked at Os 5 under 24 h/0 h day/night. The highest expression levels of *HuTIP5;1*, *HuSIP1;1*, *HuSIP1;2*, *HuNIP4;3*, and *HuNIP6;2* were observed in Os 6 under both of the two conditions. Under 24 h/0 h day/night, *HuNIP4;1* at Os 1, *HuTIP1;3* at Os 2, *HuPIP2;3*, and *HuNIP7;1* showed different expression patterns at different stages at Os 5. *HuTIP1;2* achieved its highest expression in Os 3 under 14 h/10 h day/night. The expression of *HuNIP6;1* increased from Os 1 to Os 3 and then decreased. Expression levels of *HuNIP6;1* in Os 3 and Os 4 were significantly higher than that of the other stages under 14 h/10 h day/night, suggesting that the Os 3 and Os 4 were the important periods for the flower-opening process of pitaya. However, no significant difference was detected in expression profiles of the *HuNIP6;1* under 24 h/0 h day/night. Results from expression analyses suggested that *HuNIP6;1* may be involved in the flowering-opening process of pitaya.

### 2.5. Expression Patterns of HuNIP6;1 during Flower Opening in Pitaya

**Figure 5:**
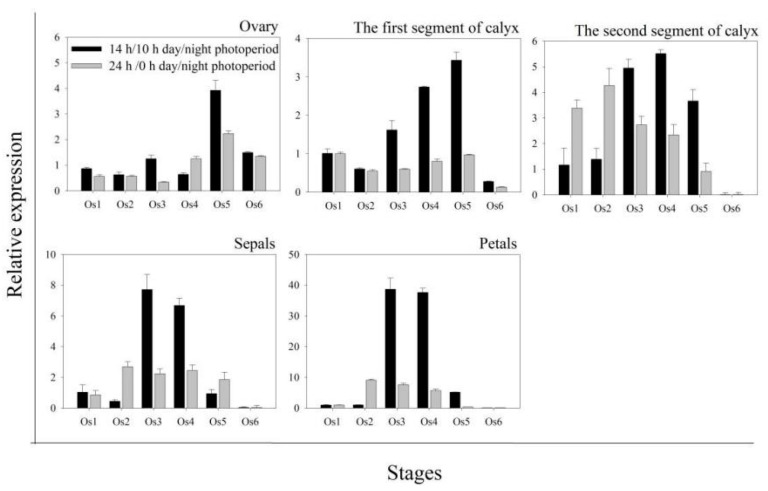
RT-qPCR analyses of *HuNIP6;1* gene in different pitaya flower sections during floral opening under 14 h/10 h day/night and 24 h/0 h day/night at 25 °C. Data represent average values from three biological replicates (±S.D.).

The expression patterns of *HuNIP6;1* were further analyzed in various tissues (ovaries, petals sepals, and the first and second segments of calyx) by RT-qPCR (Figure 5). Higher expression levels of *HuNIP6;1* were detected at Os 3 in the petals and sepals, Os 5 in ovary, and the first segment of calyx under 14 h/10 h day/night. However, *HuNIP6;1* showed higher expression levels at Os 2 in the second segment of calyx, petals, and sepals under 24 h/0 h day/night. *HuNIP6;1* was upregulated from Os 3 to Os 4 in all tissues except for the sepals under 14 h/10 h day/night, compared to down-regulation from Os 5 to Os 6 in all tissues at the two conditions.

### 2.6. Expression Pattern of HuNIP6;1 in Diurnal Cycle in Pitaya under 14 h/10 h Day/Night

**Figure 6:**
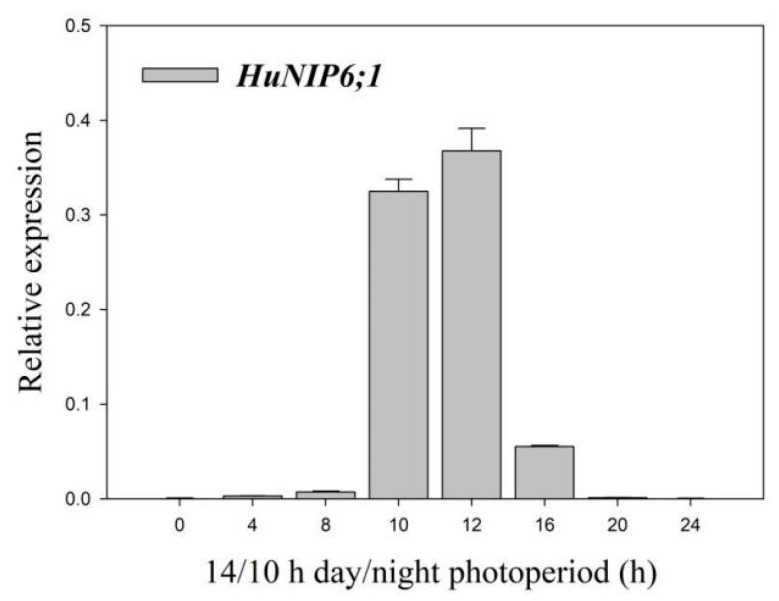
Expression levels of *HuNIP6;1* in the diurnal cycle in the pitaya petal under 14 h/10 h day/night. Data represent average values from three biological replicates (±S.D.). 12:00 on the day of flowering was supposed as the 0 h.

Circadian rhythm is synchronized with the circadian cycle, and plants contain a circadian clock that can coordinate internal and external cues to adapt to the environment. The expression patterns of *HuNIP6;1* in the diurnal cycle were analyzed in petals by RT-qPCR (Figure 6). During the flower opening of pitaya, the expression levels of *HuNIP6;1* increased from 0 h and reached its maximum at 12 h, and decreased thereafter. The results were consistent with the process of pitaya flower opening.

### 2.7. Subcellular Localization of HuNIP6;1

**Figure 7:**
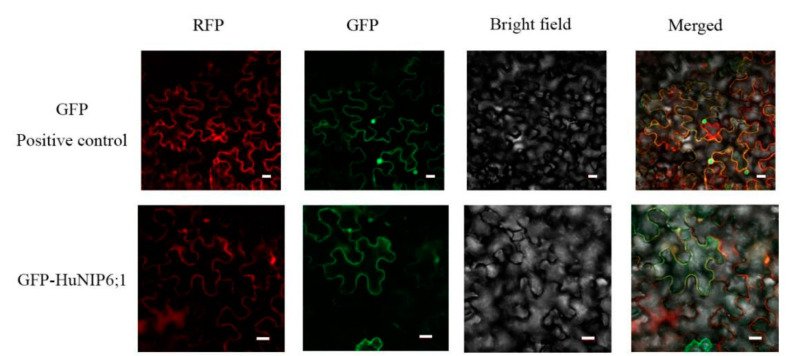
Subcellular localization of *HuNIP6;1* in epidermal cells of *Nicotiana benthamiana*. Bars = 20 μm.

Subcellular localization of *HuNIP6;1* was analyzed using transient expression of constructs in epidermal cells of *N. benthamiana.* The fluorescence for *HuNIP6;1* was apparently localized in the cell membrane, while the GFP signal for the positive control was observed in both the cell membrane and nucleus (Figure 7).

### 2.8. Over-Expression of HuNIP6;1 in Arabidopsis thaliana

**Figure 8:**
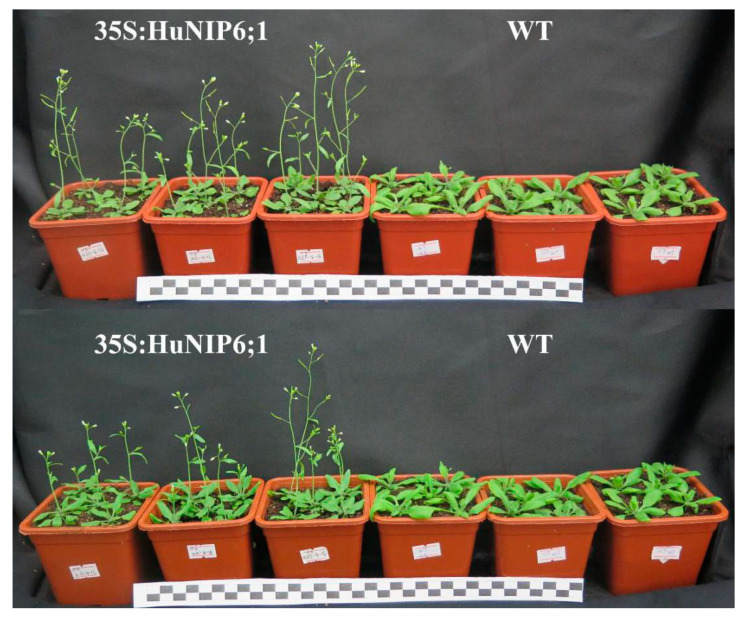
Overexpression of *HuNIP6;1* in *Arabidopsis thaliana* caused significant early flowering.

**Figure 9:**
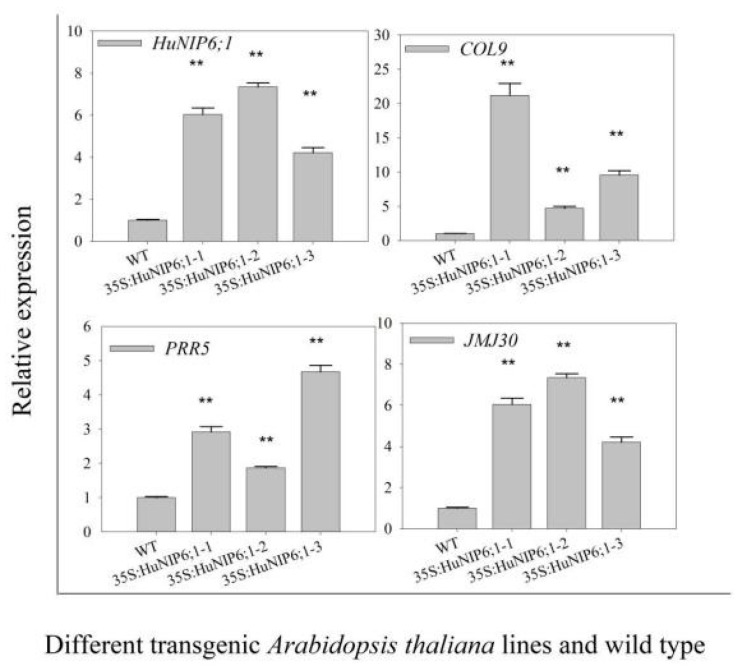
Expression levels of *HuNIP6;1* in different transgenic *Arabidopsis thaliana* lines and wild-type. Data represent average values from three biological replicates (±S.D.). Asterisks indicate significant differences (** *p* < 0.01) between the transgenic lines and wild type.

To further confirm the role of *HuNIP6;1*, we generated transgenic *A. thaliana* plants constitutively expressing *HuNIP6;1* driven by the 35S promoter (Figure 8 and Figure 9). Six different transgenic lines were obtained. Over-expression of *HuNIP6;1* in *A. thaliana* plants resulted in early flowering. The flowering time of 35S::HuNIP6;1 transgenic plants was significantly earlier than that of wild-type. The transgenic lines had higher bolting height, as well as more flower buds, fruit pods, rosette leaves, and bolting (Table 2). The expression levels of *HuNIP6;1*, *MJM30*, *COL9*, and *PRR5* in the transgenic lines were significantly higher than that of wild-type. However, no significant changes of positive flowering regulators such as *FT*, *FD*, *CO*, *LFY*, and *SCO1* were detected in transgenic *HuNIP6;1 Arabidopsis* (data not shown). Those results suggested that *HuNIP6;1* has significant functions in flowering of transgenic *A. thaliana* through regulating the other flowering-related genes.

## 3. Discussion and Conclusions

AQPs are crucial membrane transport proteins participating in the transport of water and/or small neutral solutes in plants. AQPs family members have been identified in plant species such as *A. thaliana* [3], *Brassica oleracea* [35], Zea mays [4], *B. vulgaris* [5], and *C. lanatus* [6]. In this study, the genome-wide identification and characterization of pitaya *AQPs* (*HuAQPs*) was performed using available genomic sequence data (http://www.pitayagenomic.com/) (Accessed on 15, July, 2021). The evolutionary relationships, gene structures, exon–intron distribution, conserved motifs, chromosomal distribution, and expression patterns of *HuAQPs* were analyzed. After excluding incomplete ORFs without MIP domain and NPA motifs, a total of 33 *HuAQP* genes (10 PIPs, 13 TIPs, 8 NIPs, and 2 SIPs) distributed in 9 chromosomes were identified. All groups contained *HuAQP* genes, except for XIP group. The XIP group contained one gene from *D. caryophyllus* and *B. vulgaris* [5,29]. Similar results were reported in chickpea, *Medicago*, and *Lotus japonicus* [36,37]. The number of exons in most *HuAQP* genes within PIP, TIP, NIP, and SIP was four, three, five, and three, respectively, suggesting that *HuAQPs* were highly conserved. A similar exon–intron structure is found in the other plants such as sweet orange [38], *M. truncatula* [39], watermelon [6], and longan [40].

Petals were the crucial to the flower opening and closure of *Nymphaea caerulea* [41]. The growth of carnation petals is controlled by coordinated gene expression during the progress of flower opening [42,43]. AQPs play an important role in the expansion of petal cells [17,26,42]. *Rh-PIP2;1*, a rose *AQP* gene, is involved in ethylene-regulated petal expansion. After silencing of *Rh-PIP2;1* in rose flowers, petal expansion was greatly inhibited [26]. *DcaPIP1;3*, *DcaPIP2;2*, *DcaPIP2;5*, *DcaTIP1;4*, and *DcaTIP2;2* are considered to be involved in flower-opening stages in carnation [29]. In this study, the similar gene structure and the conserved motif were found in the same subfamilies; however, their expression patterns were different (Figure 4), suggesting that different members of the same subfamily played different roles during pitaya flowering. The expression of *HuPIP1;1*, *HuPIP1;2*, *HuPIP1;4*, *HuPIP1;5*, *HuPIP2;1*, *HuPIP2;2*, and *HuPIP2;5* showed increasing patterns, and then decreased during pitaya flower opening under 14 h/10 h day/night, indicating they may play roles in flower opening. *HuTIP5;1*, *HuSIP1;1*, *HuSIP1;2*, *HuNIP4;3*, and *HuNIP6;2* had preferential expressions in the Os 6 (periods after flowering) under the two conditions, indicating that they may be involved in flower withering.

*HuNIP6;1*, a novel AQP gene located in Chr 3, was obtained from the genome data of pitaya. The length of the *HuNIP6;1* protein was 304 AAs, which is similar to other plants, such as 306 AAs in *B. vulgaris* [5] and 307 AAs in *D. caryophyllus* [29]. Results from expression analyses suggested that *HuNIP6;1* may be involved in the process of pitaya flowering. The subcellular localization showed that *HuNIP6;1* is located at the cell membrane. Over-expression of *HuNIP6;1* in *A. thaliana* significantly resulted in early flowering (Figure 8 and Figure 9). *MJM30*, *COL9*, and *PRR5* were negative flowering regulators [44,45,46]. In the present study, over-expression of *HuNIP6;1* in *Arabidopsis* significantly prompted expression of *MJM30*, *COL9*, and *PRR5.* Those results suggested that the over-expression of *HuNIP6;1* broke the original cycle by regulating the expression of *MJM30*, *COL9*, and *PRR5* to a normal rhythm in *Arabidopsis*. However, the mechanism of interaction between *HuNIP6;1* and the other flowering-related genes is unknown, and further study is necessary to elucidate it.

In summary, our study provides the first genome-wide analysis of the *AQP* family in pitaya. A total of 33 *HuAQP* genes were identified and divided into four subfamilies. A novel *AQP* gene; i.e., *HuNIP6;1*, belonging to a member of the Group NIP family, was obtained, and was located at the cell membrane. Over-expression of the *HuNIP6;1* gene in *A. thaliana* resulted in a significant early flowering, suggesting that *HuNIP6;1* plays a significant role in pitaya flowering. The results of the present study provide valuable information for a better understanding of AQPs during the flower-opening process of pitaya.

## 4. Materials and Methods

### 4.1. Sequence Analysis

Multiple sequence alignment of the amino acid sequences was performed using DNAMAN software (LynnonBiosoft, San Ramon, CA, USA) and TMHMM (server version 2.0, http://www.cbs.dtu.dk/services/TMHMM/). Amino acid number, molecular weights, and theoretical pI were analyzed on the ExPASy website (http://web.expasy.org/potparam/).

### 4.2. Phylogenetic Analysis

The full-length amino acid sequences of AQPs were downloaded from TAIR (https://www.arabidopsis.org/), sugar beet genome RefBeet-1.2 (http://bvseq.molgen.mpg.de/Genome/Download/index.shtml), and Carnation DB (http://carnation.kazusa.or.jp/index.html). A maximum-likelihood phylogenetic tree was constructed using MEGAX software with a bootstrap test conducted 1000 times [47]. The phylogenetic tree was annotated with EVOLVIEW (http://120.202.110.254:8280/evolview).

### 4.3. Gene-Structure Analysis and Identification of Conserved Motifs

TBtools software (http://www.tbtools.com/) was used for the exon–intron structure analysis, and the conserved protein motifs were analyzed using the MEME (http://meme-suite.org/) website.

### 4.4. Chromosomal Distribution

The location information of the AQPs was obtained from the pitaya genome database (http://www.pitayagenomic.com/) (Accessed on 15 July 2021). The gene-location map was constructed using MapChart [48].

### 4.5. Plant Materials

**Figure 10:**
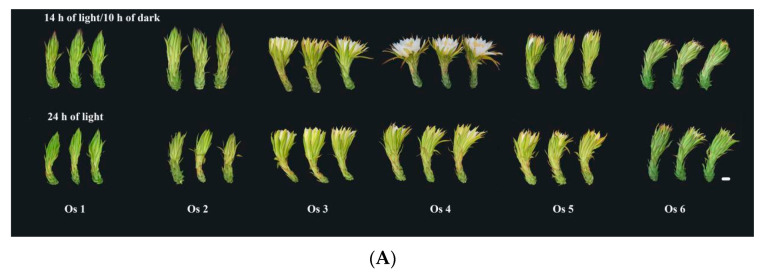

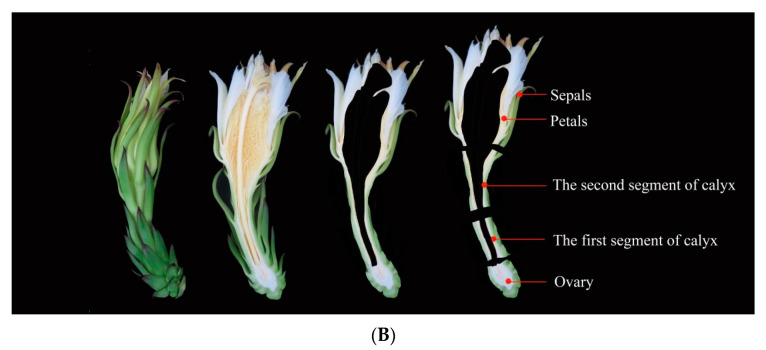
Different flower opening stages and sections of ‘Hongguan No. 1′ pitaya. (**A**) Different flower-opening stages of ‘Hongguan No. 1′ pitaya under 14 h/10 h day/night and 24 h/0 h day/night at 25 °C (n = 9, bars = 4 cm). The flower-opening process was separated into 6 stages, including opening stage 1 (Os 1), 12 h after culture (periods before flowering); Os 2, 24 h after culture (periods before flowering); Os 3, 28 h after culture (periods during flowering); Os4, 32 h after culture (periods during flowering); Os 5, 36 h after culture (periods after flowering); and Os 6, 40 h after culture (periods after flowering). (**B**) Different sections of ‘Hongguan No. 1′ pitaya flower. Bar = 4 cm.

The flowers of ‘Hongguan No. 1′ pitaya (*H. monacanthus*) were collected from Baiyun District, Guangzhou City, Guangdong Province, China. Flowers with 25 cm stems were picked on the 15th day after the flower bud formation in July and cultured at two different conditions: 14 h/10 h day/night and 24 h/0 h day/night at 25 °C. Samples were collected every 4 h from 20:00 on the same day (supposed as the 0 h). The flower-opening process was separated into six stages, including opening stage 1 (Os 1), 12 h after culture (periods before flowering); Os 2, 24 h after culture (periods before flowering); Os 3, 28 h after culture (periods during flowering); Os4, 32 h after culture (periods during flowering); Os 5, 36 h after culture (periods after flowering); and Os 6, 40 h after culture (periods after flowering) (Figure 10A). Ovaries, petals, sepals, and the first and second segments of calyx were collected for gene cloning and expression analyses (Figure 10B). To analyze the rhythmic expression of *HuNIP6;1* in pitaya petal, the samples were collected every 4 h from 12:00 on the day of flowering (supposed as the 0 h). Three uniformly sized flowers from every period were sampled as three replicates. All samples were immediately frozen in liquid nitrogen and stored at –80 °C until use.

### 4.6. RNA Isolation, cDNA Synthesis and RT-qPCR Analyses

RNA was isolated using the EASYspin Plus Plant Quick RNA isolation Kit (RN38) (Aidlab Biotechnology, Beijing, China) according to the manufacturer’s instructions. The first strand cDNA was synthesized using the PrimeScript™ RT Reagent Kit with gDNA Eraser (TaKaRa, Shiga, Japan). The primers of RT-qPCR were designed by BatchPrimer3v1.0 (http://batchprimer3.bioinformatics.ucdavis.edu/index.html) and the *Actin* (1) reference gene was used as the internal control [49]. The primers used for RT-qPCR are listed in Table S1. RT-qPCR was carried out using a LightCycler 480Ⅱ real-time PCR system (Roche, Switzerland) using the RealUniversal Color PreMix (SYBR Green) (TIANGEN, Beijing, China) according to the manufacturer’s instructions. Three biological replicates were performed for each sample. The relative expression levels were calculated using the comparative 2^−△△C^T method [50].

### 4.7. Cloning of HuNIP6;1 Gene

*HuNIP6;1* was cloned using specific primers of ATGGATGCTGAGGATCCCGG and TCATCTCCGGAAGCTTGGTG following the procedure of Xie et al. [51].

### 4.8. Subcellular Localization of HuNIP6;1

The full-length coding sequence of *HuNIP6;1* was inserted into the pCAMBIAL1300-eGFP vector. *Agrobacterium tumefaciens* GV3101 cells carrying 35S-HuNIP6;1-eGFP or GFP-positive control were infiltrated into tobacco (*Nicotiana benthamiana*) leaves, respectively. Imagines were viewed with a confocal laser microscope (ZEISS LCM-800, Oberkochen, Germany).

### 4.9. Arabidopsis thaliana Transformation and Phenotypic Analysis

The full-length of *HuNIP6;1* was subcloned into the pPZP6k90 vector under the control of the 35S promoter and transformed into *A. thaliana* by the *Agrobacterium tumefaciens*-mediated method. The 35S:HuNIP6;1 transformed lines were identified by PCR detection, and the expression levels of *HuNIP6;1* were analyzed by RT-qPCR. The phenotypes of T_4_ homozygous transgenic plants were analyzed and photographed with a digital camera (G16, Canon).

## Figures and Tables

**Table 1:** Characteristics of the 33 *HuAQP* genes in pitaya.

Family	Gene Names	GenBank No.	Chromosomal Distribution	ORF (bp)	Length (AA)	pI	Molecular Weight (kDa)
PIP	*HuPIP1;1*	*HU04G00221.1*	4	858	285	9.13	30.51
	*HuPIP1;2*	*HU05G01480.1*	5	855	284	9.16	30.40
	*HuPIP1;3*	*HU06G00483.1*	6	858	285	9.11	30.48
	*HuPIP1;4*	*HU05G00658.1*	5	858	285	8.84	30.62
	*HuPIP1;5*	*HU11G01779.1*	11	858	285	9.19	30.75
	*HuPIP2;1*	*HU01G00026.1*	1	858	285	9.1	30.41
	*HuPIP2;2*	*HU03G00407.1*	3	852	283	8.98	30.20
	*HuPIP2;3*	*HU06G02121.1*	6	861	286	9.08	30.58
	*HuPIP2;4*	*HU09G00753.1*	9	861	286	8.59	30.53
	*HuPIP2;5*	*HU06G02083.1*	6	861	286	7.66	30.72
TIP	*HuTIP1;1*	*HU09G00676.1*	9	768	255	5.84	26.22
	*HuTIP1;2*	*HU06G01671.1*	6	756	251	6.09	26.03
	*HuTIP1;3*	*HU09G01283.1*	9	726	241	7.69	24.54
	*HuTIP1;4*	*HU02G01483.1*	2	753	250	5.35	25.74
	*HuTIP2;1*	*HU05G02336.1*	5	732	243	6	24.42
	*HuTIP2;2*	*HU05G00450.1*	5	732	243	6	24.42
	*HuTIP2;3*	*HU04G00025.1*	4	702	233	5.1	23.45
	*HuTIP2;4*	*HU03G00687.1*	3	750	249	5.76	25.29
	*HuTIP2;5*	*HU01G00787.1*	1	744	247	5.59	25.28
	*HuTIP3;1*	*HU08G00860.1*	8	774	257	7.1	27.27
	*HuTIP4;1*	*HU02G02913.1*	2	762	253	6.36	26.28
	*HuTIP4;2*	*HU02G02914.1*	2	762	253	6.57	26.30
	*HuTIP5;1*	*HU06G02398.1*	6	771	256	8.74	26.25
NIP	*HuNIP4;1*	*HU08G01901.1*	8	882	293	9.51	31.12
	*HuNIP4;2*	*HU05G01754.1*	5	906	301	7.7	32.23
	*HuNIP4;3*	*HU06G00624.1*	6	840	279	6.82	29.64
	*HuNIP4;4*	*HU05G01185.1*	5	867	288	9.48	30.34
	*HuNIP5;1*	*HU06G00253.1*	6	933	310	8.71	31.98
	*HuNIP6;1*	*HU03G00523.1*	3	915	304	6.07	31.43
	*HuNIP6;2*	*HU05G00189.1*	5	654	217	7.11	22.72
	*HuNIP7;1*	*HU08G00592.1*	8	864	287	8.3	29.78
SIP	*HuSIP1;1*	*HU09G01607.1*	9	780	259	9.33	27.16
	*HuSIP1;2*	*HU02G03098.1*	2	744	247	9.63	26.20

**Table 2:** Phenotype of wild-type and 35S:HuNIP6;1 transgenic lines.

	Bolting Height (cm)	No. of Flower Buds	No. of Fruit Pods	No. of Rosette Leaves	No. of Bolting
Wild-type	0.90 d	4.0 e	0 c	8.0 d	0 e
35S:HuNIP6;1 transgenic line 1	12.57 a	19.0 ab	3.0 a	8.0 cd	2.0 bcd
35S:HuNIP6;1 transgenic line 2	8.74 b	18.0 b	1.0 c	10.0 a	2.0 cd
35S:HuNIP6;1 transgenic line 3	9.27 b	21.0 a	2.0 b	9.0 ab	3.0 a
35S:HuNIP6;1 transgenic line 4	6.30 c	12.0 d	0 c	9.0 bcd	2.0 d
35S:HuNIP6;1 transgenic line 5	5.21 c	14.0 cd	0 c	9.0 bc	3.0 b
35S:HuNIP6;1 transgenic line 6	8.27 b	15.0 c	0 c	9.0 cd	3.0 bc

The data are presented as means (n = 20). Different letters within the same column indicate a significant difference at the 5% level according to Duncan’s multiple range test.

## Data Availability

Data is contained within the article and Supplementary Material.

## Supplementary Materials

The following are available online at https://www.mdpi.com/article/10.3390/ijms22147689/s1.

## Abbreviations

AA	Amino acid
AQP	Aquaporin
CCA1	Circadian clock associated 1
cDNA	Complementary DNA
CO	Constans
COL9	Constans-like 9
FD	Flowering locus D
LFY	LEAFY
FT	Flowering locus T
GFP	Green fluorescent protein
eGFP	Enhance green fluorescent protein
kDa	Kilodaltons
LHY	Late elongated hypocotyl
MJM	Jumonji-C domain-containing protein
NCBI	National Center for Biotechnology Information
ORF	Open reading frame
pI	Theoretical isoelectric
PRR	Pseudo response regulator
RT-qPCR	Reverse transcription quantitative real-time polymerase chain reaction
SOC1	Suppressor of overexpression of constans 1
TOC1	Timing of CAB expression 1
