# Supplementary material for: Genome-Wide Identification of Aquaporin Gene Family in Pitaya Reveals an HuNIP6;1 Involved in Flowering Process
Source: Int J Mol Sci. 2021 Jul 19;22(14):7689. doi: 10.3390/ijms22147689 (PMC8306030; doi:10.3390/ijms22147689)
Supplement: Supplementary file 1 [file ijms-22-07689-s001.zip › ijms-1291929-supplementary.pdf]

Supplementary Table S1

Primers of genes for qRT-PCR

Gene names	Forward primer (5'-3')	Reverse primer (5'-3')
HuPIP1;1	AAACCCAGCAGTGACCTTTG	CCACCCAACACTTCATAGGG
HuPIP1;2	CATGGTAATGCAGTGCTTGG	ACGAAGGTGCCAACAATCTC
HuPIP1;3	ACACTGTCTTCTCCGCCACT	GACTCCTGGCTGGGTTGATA
HuPIP1;4	GCACATAAACCCAGCAGTGA	CTCCACCACCCAACATCTG
HuPIP1;5	GAGATTGTGGGCACCTTTGT	ATAGGGATGGTTGCCAAGTG
HuPIP2;1	TCTAAGGAGGTGAGCGAGGA	ATGAACTCGGCAATGAGAGC
HuPIP2;2	CACCCTCATGGGAAGGACTA	GGCGATGGTAACGTAGAGGA
HuPIP2;3	GCACATAAACCCAGCAGTGA	CCATAGCGGTCGTAGTAGGC
HuPIP2;4	GTGACATTCGGGCTGTTCTT	CCTCCGCCGTACCTATTGTA
HuPIP2;5	GCATCAACCCTGCTCGTAGT	CCTTGACAAAGCTGGCTCTC
HuTIP1;1	ACTGGTGGCTTGGAAACATC	TTGGCTCCGACAATGAAAC
HuTIP1;2	TTGACCCAAAGAAAGGCAAC	CCGAGCCAATAGACCCAGT
HuTIP1;3	GGCTGAGTTCATTTCCATGC	TGAGACAGCCACAAACAAGG
HuTIP1;4	GGCTGAGTTCATTTCCATGC	TGAGACAGCCACAAACAAGG
HuTIP2;1	TGCTTGCCTTCTCCTCAAAT	TGTGCCCTTCTTAGGGTCAG
HuTIP2;2	TGCTTGCCTTCTCCTCAAAT	TGTGCCCTTCTTAGGGTCAG
HuTIP2;3	TGCTGGTGTAGGATCTGCTG	AGCTGGGTTCAAATGTCCAC
HuTIP2;4	GTCTCCGTGGGTGCTAACAT	AAGGAAACAAGCGACAATGG
HuTIP2;5	GCTGGTAGGAGTGGCAGTGT	AGACGCCAGTTAGGATGGTG
HuTIP3;1	ATGGCGCCGCCTCGGAGATA	TTAGTAATCTTCTGGTGCCAAAGGTTG
HuTIP4;1	CAAAGAAAGGCCCACTTCAA	GATCCAGTGATCGGTCCAGT
HuTIP4;2	CAAAGAAAGGCCCACTTCAA	AGATCCAGTGATCGGTCCAG
HuTIP5;1	TGGCCTCGGCTGTCTATATC	GAGGCAAGCCATAACAGAGC
HuNIP4;1	TGATATTTGCAGGGTGTGGA	ACAGCAGGGTTGAAATGAGC
HuNIP4;2	CAATGAACCCAGCAAGGAGT	AGTTTGGATGGCAACTGGTC
HuNIP4;3	GCTCAGTGGTGGTGAACAAG	AAGAGTAACGGCAGGGTTGA
HuNIP4;4	ATTTCCCTTGGAAGCAGGTT	GCTTGAACATCTGTGCCTGA
HuNIP5;1	TTTCATGTCAGGCGGTGTAA	CTCCCACTGCAATACCTGCT
HuNIP6;1	TTTGGAGGATTTAGCGGAGA	TAGAGGGACAGAGGGAGGTG
HuNIP6;2	ATCCGGTGAGGACACTAGGA	GACAGCATTGTGGATGTTGG
HuNIP7;1	TGCTCGCAACATTTATGGTC	GATTCAACGACCCTCCAGAA
HuSIP1;1	AGCCATCTTAGAAGCGATGC	AGCTCGAATCCTGTGGGTAA
HuSIP1;2	TGGCAGTCCTTGTCATCATC	ACATAAGCCCATCCAAATGC
Actin(1)	AAAGGCTAACAGGGAGAAAA	GACCACTGGCGTAAAGAGAA
JMJ30	GTATGCTTCTCCGCAAGGA	CGGGACCAATCTCTCACCT
COL9	AGCAGCATAAGAGGCAAACC	CTTGTCGGTAGGACCATCATC
PRR5	GCCAAAGAGAGGTGACTAATGG	TCGGAGGTGAGGGTTGCTA
